# Lyophilization protects [FeFe]-hydrogenases against O_2_-induced H-cluster degradation

**DOI:** 10.1038/srep13978

**Published:** 2015-09-14

**Authors:** Jens Noth, Ramona Kositzki, Kathrin Klein, Martin Winkler, Michael Haumann, Thomas Happe

**Affiliations:** 1 Ruhr-Universität Bochum, Fakultät für Biologie und Biotechnologie, Lehrstuhl für Biochemie der Pflanzen, AG Photobiotechnologie, 44801 Bochum, Germany; 2 Freie Universität Berlin, Institut für Experimentalphysik, 14195 Berlin, Germany; 3 Ruhr-Universität Bochum, Fakultät für Chemie und Biochemie, Anorganische Chemie I–Bioanorganische Chemie, 44801 Bochum, Germany

## Abstract

Nature has developed an impressive repertoire of metal-based enzymes that perform complex chemical reactions under moderate conditions. Catalysts that produce molecular hydrogen (H_2_) are particularly promising for renewable energy applications. Unfortunately, natural and chemical H_2_-catalysts are often irreversibly degraded by molecular oxygen (O_2_). Here we present a straightforward procedure based on freeze-drying (lyophilization), that turns [FeFe]-hydrogenases, which are excellent H_2_-producers, but typically extremely O_2_-sensitive in solution, into enzymes that are fully resistant against O_2_. Complete dryness protects and conserves both, the [FeFe]-hydrogenase proteins and their inorganic active-site cofactor (H-cluster), when exposed to 100% O_2_ for days. The full H_2_-formation capacity is restored after solvation of the lyophilized enzymes. However, even minimal moisturizing re-establishes O_2_-sensitivity. The dry [FeFe]-hydrogenase material is superior also for advanced spectroscopic investigations on the H-cluster reaction mechanism. Our method provides a convenient way for long-term storage and impacts on potential biotechnological hydrogen production applications of hydrogenase enzymes.

The global market for enzymatically catalyzed reactions and their products is continuously growing. The use of enzymes often is preferable over conventional chemical processes because of their stereo-selective chemistry and superior turnover rates under moderate temperatures and pressures. With the large-scale cultivation of bacteria and yeast as homologous and heterologous expression hosts, valuable products can be generated in large and therefore economically attractive quantities. Besides the industrial production of metabolites like vitamins, amino acids, vaccines or antibiotics a number of highly active and specific enzymes are produced and purified for the *in vitro* generation of further high-value products and applied in food-, pharmaceutical-, and detergent-industry^1^. Also the production of second-generation biofuels by enzymatic polymer-degradation using cellulases and of first-generation biofuels like ethanol and methane from biomass fermentation are commercially well established procedures^2^.

A promising fuel resource that can also be produced biologically is molecular hydrogen (H_2_). Two H_2_-producing biocatalysts are found in nature, which inspire modern biomimetic catalyst research. Nitrogenases turn molecular nitrogen into ammonia, a process which yields H_2_ only as a byproduct^3^. On the other hand, hydrogenase enzymes directly catalyze the reduction of protons to form H_2_ as the primary product^4^,^5^. With reported turnover rates of up to 10,000 molecules of H_2_ per second, [FeFe]-hydrogenases are the most efficient known biocatalysts for H_2_ generation and therefore promising targets for potential applications^6^,^7^,^8^.

The catalytically active cofactor (H-cluster) of [FeFe]-hydrogenases is a [6Fe6S] complex (further on denoted [6FeH]), consisting of a standard cubane [4Fe4S] cluster ([4FeH]) and a unique diiron complex ([2FeH]) which represents the actual active site of reversible H_2_-production^8^,^9^,^10^. The two iron atoms in [2FeH], in distal (Fe_d_) or proximal (Fe_p_) positions to [4FeH], bind unusual carbon monoxide (CO) and cyanide (CN^-^) ligands, which tune the electrochemical and redox properties of the H-cluster and guide state transitions during the catalytic cycle^11^,^12^. An azadithiolate (adt = S_2_(CH_2_)_2_NH) ligand connects the two iron ions in [2FeH] and its secondary amine bridgehead shuttles protons between a conserved H^+^-transfer pathway in the protein and the site of catalytic turnover^13^,^14^,^15^. The sub-clusters in [6FeH] are covalently and electrochemically coupled through one of the four cysteine ligands that bind the [4FeH] complex to the protein scaffold^16^.

A very high hydrogenase activity and thus H_2_-production yield can be achieved *in vitro* using purified [FeFe]-hydrogenase protein when the natural electron mediators are substituted by redox-active chemicals or the enzyme is immobilized on an electrode^7^,^17^,^18^. Under such conditions, the rate-limiting metabolic pathways of electron supply as present in the living cell are omitted and competitive redox processes are absent^6^,^18^,^19^. The major obstacle for introducing these promising H_2_-producing biocatalysts into biotechnological applications, however, is their sensitivity towards molecular oxygen (O_2_). While all hydrogenases are inhibited by O_2_ to a certain extent, [FeFe]-hydrogenases exhibit the highest level of O_2_-sensitivity due to irreversible degradation of the H-cluster within seconds to minutes during exposure even to traces of dioxygen^20^,^21^,^22^.

In this study, we present a method which prevents the O_2_-induced inactivation of two [FeFe]-hydrogenases representing different prototypic enzyme structures. HYDA1 from the green alga *Chlamydomonas reinhardtii* represents the minimal M1-type, binding only the H-cluster, whereas CPI from the bacterium *Clostridium pasteurianum* represents the most complex monomeric M3-type, containing in addition to the H-cluster three [4Fe4S] and one [2Fe2S] cluster^23^. We developed and applied a technique for protein lyophilization (freeze-drying by water sublimation at low pressure and cryogenic temperatures^24^), which removes bulk and protein-bound water from protein preparations, respectively, for the preservation of [FeFe]-hydrogenases. Our biochemical analyses show that these extremely O_2_-sensitive enzymes after lyophilization are completely protected against O_2_. Structural characterization using X-ray absorption spectroscopy (XAS)^25^,^26^,^27^,^28^ on the lyophilized proteins revealed the integrity of the H-cluster. The procedure of freeze-drying thereby is fully reversible, yet also restoring O_2_-sensitivity in solution. We suggest that the H-cluster degradation requires both, O_2_ attack and either specific protein bound H_2_O molecules or at least a water-dissolved enzyme state. These findings are an important milestone for biotechnological use of [FeFe]-hydrogenases as sustainable catalysts for H_2_ fuel generation and provide a new impulse in the challenging task to overcome the enzyme’s Achilles heel.

## Results

### Improved purification yields of HYDA1 [FeFe]-hydrogenase

Significant improvements in purification yield and specific activity have been achieved for [FeFe]-hydrogenase HYDA1 from *C. reinhardtii* since the first isolation about 30 years ago (Supplementary Tab. S1)^29^. Here, besides HYDA1 a second [FeFe]-hydrogenase type, CPI from *C. pasteurianum,* was produced by overexpression in *Escherichia coli*, which contained the active-site cofactor in two different maturation states. The inactive proteins denoted [4FeH] bind only the [4Fe4S] subsite of the H-cluster that is assembled by the iron-sulfur cluster housekeeping (ISC) machinery in *E. coli,* whereas the fully active holoenzyme denoted [6FeH] is equipped with both, the four-iron and the diiron ([2FeH]) sub-complexes of the H-cluster. In the living host, synthesis and assembly of the [2FeH]-subsite requires specific maturases, namely HYDE, HYDF and HYDG^30^,^31^. By replacing the previously used Strep-tag II (STII) with a hexahistidine-tag (HIS_6_) fused to the HYDA1 protein, we improved the yield of HYDA1[6FeH] expressed in *E. coli* containing the HYDE,F,G background by a factor of 1.5 and the yield of HYDA1[4FeH] expressed in *E. coli* without HYDE,F,G by a factor of 3 (Supplementary Fig. S1; Supplementary Tab. S1) in comparison to previously reported purification procedures^32^. An efficient method for *in vitro* H-cluster maturation has been recently reported, in which the [4FeH] protein is activated with a chemically synthesized analogue (here denoted 2Fe_adt_) of the diiron site, thereby forming the [6FeH] enzyme with full activity^14^. The combination of the HIS_6_-tagged HYDA1[4FeH] construct and *in vitro* maturation with 2Fe_adt_ thus facilitated the production of up to 90 mg of fully active HYDA1[6FeH] with a typical specific activity of about 950** **µmol H_2_ · mg^-1^ · min^-1^ per liter of *E. coli* culture (Supplementary Tab. S1)^14^. This amount of enzyme is capable to produce about 2 liters of H_2_ per minute under maximum turnover conditions.

### Lyophilization of two different [FeFe]-hydrogenases

**Figure 1:**
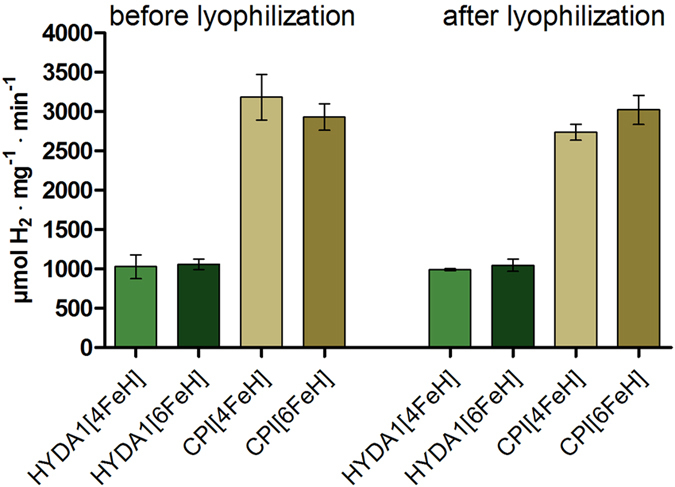
Specific H_2_-evolution activities of HYDA1 and CPI before and after lyophilization. Specific H_2_-production rates of the [FeFe]-hydrogenases HYDA1 (green) and CPI (brown) were determined using the sodium-dithionite-driven methyl-viologen-reduction assay (1** **μg HYDA1 or 0.5** **μg CPI). The enzymes were both assayed before or after lyophilization and subsequent re-suspension; in addition, [4FeH]-proteins were maturated with 2Fe_adt_ prior to activity determination. Mean activities from at least 3 independent measurements are shown, error bars give the standard deviations.

The process of lyophilization, also called freeze-drying, is commonly used to preserve valuable and labile molecules for application^33^,^34^. HYDA1 and CPI proteins in the [4FeH] and [6FeH] maturation states were first deep-frozen in liquid nitrogen and then dehydrated under vacuum through water sublimation, resulting in lyophilized samples. Thereafter, the H_2_-evolution activity was assayed after re-solvation of aliquots of the dry proteins in buffer solutions. The lyophilized [6FeH]-containing holoenzymes, *in vitro* maturated with the 2Fe_adt_ complex prior to lyophilization, were assayed directly after rehydration. Freeze-dried hydrogenases, which only contained the [4FeH] were *in vitro* activated during rehydration with a 10-fold molar excess of 2Fe_adt_ to yield active [6FeH]-containing enzymes after lyophilization. In addition, the enzyme activities of non-lyophilized (control) [6FeH]- and 2Fe_adt_-activated [4FeH]-samples and lyophilized protein samples were compared (Fig. 1).

The HYDA1 and CPI preparations showed a 1:3 ratio of their specific activities, which is in agreement with previous results^14^. No significant differences were found between the H_2_-evolution rates of previously purified 2Fe_adt_-activated [6FeH]-enzymes and [4FeH]-enzymes that were *in vitro* activated immediately prior to the assay. The specific H_2_-activities after lyophilization were practically identical to the activities determined for the non-lyophilized control samples for both HYDA1 and CPI, demonstrating the complete reversibility and activity recovery after rehydration of the lyophilized [FeFe]-hydrogenases. These results reveal that both, chlorophycean M1-type HYDA1 and bacterial M3-type CPI [FeFe]-hydrogenases can be lyophilized without any significant loss of catalytic activity (Fig. 1). Accordingly, the feasibility and reversibility of lyophilization is independent from the level of structural complexity of the [FeFe]-hydrogenase. Moreover, there was no need for additives like lyo- or cryo-protectants to shelter the proteins, because full activity recovery was achieved for enzymes lyophilized in a common storage buffer (Tris-HCl, pH 8).

### O_2_-tolerance of lyophilized [FeFe]-hydrogenases

**Figure 2:**
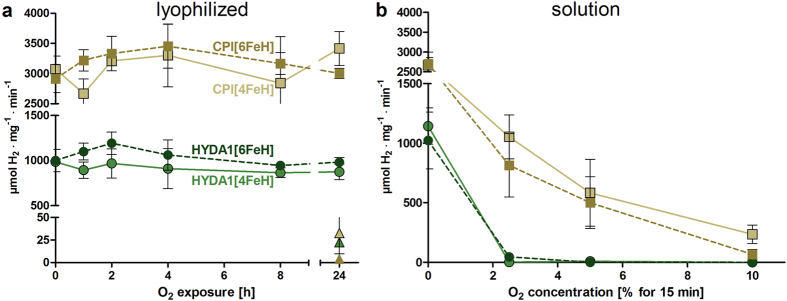
O_2_-sensitivity of lyophilized and dissolved [FeFe]-hydrogenases. Sample annotations are as in Fig. 1 and H_2_-activities were determined accordingly. Hydrogenase proteins were (**a**) exposed after lyophilization to 100% O_2_ for up to 24 hours (green symbols, HYDA1; brown symbols, CPI) or to ambient air for 24 h (triangles) at room temperature or (**b**) solutions of non-lyophilized proteins (6.33 nM) were incubated with the indicated O_2_-levels for 15 min at 37 °C. Values are the mean of at least 3 independent measurements, error bars give standard deviations.

The O_2_-sensitivity levels of control and lyophilized HYDA1 and CPI samples were compared. The lyophilized [4FeH] and [6FeH] proteins were exposed to dry pure O_2_ gas for up to one day and H_2_-evolution activities were determined after subsequent enzyme dissolution (and maturation with 2Fe_adt_ of the [4FeH] proteins) (Fig. 2a). In addition, lyophilized proteins were exposed to ambient air for 24 h. Solution samples of the non-lyophilized hydrogenases were incubated with increasing O_2_-concentrations for only 15 min for comparison (Fig. 2b).

The results show that the lyophilized hydrogenases retained their full catalytic competence even after 24 h exposure to 100% O_2_. This holds no matter whether [4FeH] or [6FeH] proteins were exposed to O_2_. The enzymes in solution, however, almost entirely lost H_2_-production activity already after 15 min under 10% O_2_ (CPI) or 2.5% O_2_ (HYDA1). The degrees of inactivation of the solution samples were similar for [6FeH] and [4FeH] proteins, meaning that not only the complete H-cluster, but also the [4FeH] cluster alone apparently is damaged by O_2_. Interestingly, all lyophilized hydrogenase proteins were almost completely inactivated after incubation in ambient air for 24 h (Fig. 2a). A plausible explanation for this behavior was that the humidity (water vapor) in the air caused partial rehydration of the proteins^35^,^36^, which restored the O_2_-sensitivity, as examined further below.

### Restoration of O_2_-sensitivity by humidity

**Figure 3:**
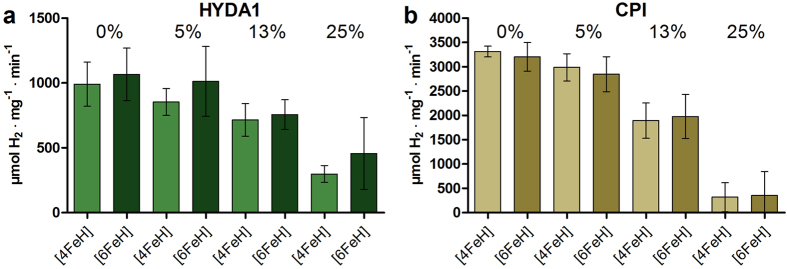
O_2_-inactivation of lyophilized [FeFe]-hydrogenases by increasing humidity. Lyophilized HYDA1 (**a**) and CPI (**b**) proteins in their [4FeH] or [6FeH] maturation states were exposed for 30 min to the indicated levels of humidity in pure O_2_ gas prior to specific activity determination. Values represent the mean of 3 independent measurements, error bars give standard deviations.

We analyzed the impact of atmospheric humidity on the O_2_-induced inactivation rate of the lyophilized [FeFe]-hydrogenases. Increasing amounts of H_2_O-saturated O_2_ gas were injected into sealed reaction vials containing lyophilized hydrogenase in an atmosphere of O_2_ and the samples were incubated at room temperature for 30 min prior to H_2_-evolution activity determination (Fig. 3). The data show that the catalytic activity of all lyophilized proteins in the presence of O_2_ was decreasing for increasing humidity levels. For CPI no difference between the activities of [4FeH] and [6FeH] proteins was observed. For HYDA1, however, the O_2_-sensitivity seems to be slightly higher for increasing humidity in the [4FeH] protein compared to the [6FeH] protein. This finding corroborated the observation described above that already the protein containing only the [4FeH] cluster is a target for O_2_ attack.

### H-cluster integrity studied by X-ray absorption spectroscopy

**Figure 4:**
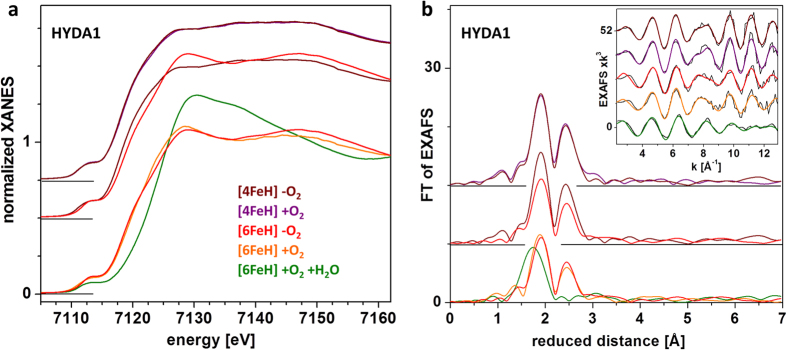
Fe-XAS analysis of lyophilized HYDA1 proteins. (**a**) XANES spectra of the indicated [FeFe]-hydrogenase samples. (**b**) Fourier-transforms (FTs of experimental data) of EXAFS spectra in the inset (black lines, experimental data; colored lines, simulations with parameters in Table 1). The color code is the same and spectra were vertically displaced for comparison in panels (**a and b**).

XAS was employed to characterize the oxidation state and molecular structure (iron-ligand bond lengths and Fe-Fe distances) of the H-cluster sub-complexes in the lyophilized HYDA1 proteins (Fig. 4). The redox level and metric parameters are indicative of the H-cluster integrity^24^. We compared the following preparations: (1) HYDA1[4FeH] protein overexpressed in *E. coli* in the absence of the three maturases HYDE,F,G was prepared anaerobically and studied in the anoxic state ([4FeH] −O_2_) and after exposure to dry pure O_2_ gas for ~15 min ([4FeH] +O_2_). (2) HYDA1[6FeH] protein, which contains the complete H-cluster was studied in the anoxic state ([6FeH] −O_2_), after exposure to dry pure O_2_ gas for ~15 min ([6FeH] +O_2_), and after exposure to air at ambient humidity for ~5 days ([6FeH] +O_2_ +H_2_O). XAS experiments on lyophilized HYDA1 proved to be highly advantageous, because the X-ray fluorescence signal intensity was increased about 10-fold compared to solution samples with similar protein contents and the scattering background was drastically diminished due to the absence of water, which largely improved the XAS data quality at shortened measuring times.

The XANES and EXAFS spectra of lyophilized HYDA1[4FeH] even after extended O_2_ exposure were very similar to the spectra of HYDA1[4FeH] in anoxic solution^28^, indicating a similar iron coordination environment and thus an intact [4Fe4S] subsite of the H-cluster under both conditions (Fig. 4a). The EXAFS analysis revealed the typical four Fe-S bonds (~2.3 Å) and three Fe-Fe distances (~2.7 Å) per Fe ion in the cubane cluster, both in the absence and after exposure to O_2_ (Tab. 1). The structure of the [4Fe4S] cluster thus was not significantly affected by lyophilization and O_2_-exposure. The XAS spectra of lyophilized anoxic HYDA1[6FeH] differed pronouncedly from the spectra of HYDA1[4FeH] (Fig. 4b), indicating quantitative binding of the [2FeH] unit to the cubane cluster in the 2Fe_adt_-maturated HYDA1[6FeH]^13^,^14^. The K-edge energy of the lyophilized HYDA1[6FeH] was similar within ±0.15 eV to that of heterologously expressed HYDA1[6FeH] from *Clostridium acetobutylicum* in solution, suggesting that not more than one Fe ion in the lyophilized protein differed by one oxidation step from the reduced state of the H-cluster (H_red_)^27^,^28^. The EXAFS was well simulated using similar iron-ligand bond lengths and Fe-Fe distances as for enzyme from *C. acetobutylicum* in solution^10^,^20^,^21^, showing that a complete and intact H-cluster was present in the lyophilized HYDA1[6FeH] (Tab. 1).

After ~15 min O_2_-exposure of lyophilized HYDA1[6FeH], the XANES revealed only small changes and the EXAFS still was compatible with an intact H-cluster (Tab. 1). However, slight shortening of the Fe-Fe distances attributable to the [4Fe4S] cluster and shortening of the Fe-C(=O/N) bonds and elongation of the Fe-Fe distance in [2FeH], as well as an increased K-edge energy and amplitude compared to the anoxic protein may be explained by one-electron oxidation of the H-cluster and possible binding of an oxygen species to [2FeH]^16^,^21^. Accordingly, O_2_-induced H-cluster degradation as observed in solution was prevented in the lyophilized HYDA1[6FeH] sample^21^. After long-term exposure to air at ambient humidity, however, the drastically altered XANES reflected symmetrization of the iron coordination in octahedral sites and the EXAFS indicated the loss of most Fe-Fe distances and extensive replacement of iron-sulfur by iron-oxygen bonds (Tab. 1)^20^,^21^. O_2_-induced H-cluster degradation thus only occurs upon rehydration of the lyophilized protein.

## Discussion

Increasing efforts have been made to stabilize O_2_-sensitive enzymes for utilization as sustainable catalysts in biotechnological applications. Several highly promising members of the enzyme class of oxidoreductases are especially prone to rapid inactivation under aerobiosis including nitrogenases and hydrogenases^20^,^21^,^37^. Oxidoreductases carry redox-active cofactors that facilitate essential electron and proton transfer steps during substrate turnover. In particular, iron-sulfur clusters are ubiquitously employed as electron transfer relays in redox enzymes and often a target of O_2_ attack^38^. Removal of harmful oxygen species from protein preparations *in vitro* may be achieved using chemical reductants or enzyme-based O_2_-scavengers. However, these approaches often have certain disadvantages, like undesired pH-changes, and therefore are not well suitable for sustained biotechnological processes^39^. Removal of O_2_ from the processing atmosphere usually requires significantly larger technical efforts than removal of water (drying).

Hydrogenases are efficient catalysts for both, the production and oxidation of H_2_ and in general rather sensitive to O_2_. Depending on the enzyme family, O_2_ induces either reversible or irreversible inactivation processes, both of which have been analyzed at the mechanistic and structural level^4^,^22^. In recent years a variety of promising strategies have been pursued to understand and increase the varying levels of O_2_-tolerance in the hydrogenases, including research on the mechanisms of the O_2_ reactions at the cofactors^20^,^21^,^40^,^41^,^42^, enzyme modifications by mutagenesis^43^,^44^, and development of indirect protection systems. A recently reported *in vitro* approach uses embedding of the enzymes in a polymer-hydrogel matrix to shelter electrode-coupled hydrogenase from O_2_-attack^19^. This system stabilized H_2_-oxidation, but not H_2_-evolution in an atmosphere with up to 7% O_2_, which, however, was still considerable lower than atmospheric O_2_ partial pressure (~21%). Electrons for the reduction of detrimental oxygen species at the active site were delivered via viologen moieties in the matrix, mimicking the function of the recently discovered unusual proximal [4Fe3S] cluster in membrane-bound [NiFe]-hydrogenases, which natively show significant O_2_-tolerance^19^,^41^,^45^,^46^,^47^.[Table t1]

The catalytic efficiency for proton reduction to H_2_ is at least ten-times larger for [FeFe]-hydrogenases compared to [NiFe]-hydrogenases^48^,^49^. An approach to generate a non-immobilized [FeFe]-hydrogenase preparation, which can be stored in a stable and catalytically intact state under aerobiosis has not been described yet. We show that lyophilization is a way to produce [FeFe]-hydrogenase material resistant to O_2_. By applying a lyophilization technique under reducing conditions, an O_2_-resistant protein was obtained for representatives of two major [FeFe]-hydrogenase sub-families, namely HYDA1 (M1-type) and CPI (M3-type). No special precautions such as the use of cryo- or lyo-protectants as described for other enzymes^35^,^50^ were necessary for the [FeFe]-hydrogenases to facilitate recovery of full catalytic activity after rehydration. Moreover, the absence of surface-exposed thiol groups, which are a major factor in oxidative damaging of many lyophilized proteins due to formation of inter- or intra-molecular disulfide bonds^51^, may prevent the generation of irreversible aggregates and activity loss in the [FeFe]-hydrogenases.

Our XAS analysis clearly shows that the structure of the [4Fe4S] cluster in HYDA1[4FeH] is not affected by lyophilization nor does the cluster react with O_2_ in the dry protein. In contrast, HYDA1[4FeH] loses its competence for *in vitro* maturation with the synthetic [2FeH] analogue 2Fe_adt_ to yield active holoenzyme when exposed to O_2_ under humid conditions. The almost identical humidity-dependences of the O_2_-induced [6FeH] holoenzyme inactivation and of the loss of the maturation competence of the [4FeH] apoenzyme show that under 100% O_2_ even the [4Fe4S] cluster in the absence of the [2FeH] site is reacting with O_2_.

For O_2_-sensitive bacterial [4Fe4S]-cluster ferredoxins it has been shown that O_2_-exposure may either lead to a relatively stable [3Fe4S] product that can be reactivated or causes complete cluster degradation^52^. The susceptibility of a cofactor to oxidative attack may correlate with its surface accessibility^53^,^54^. The vacant [2FeH] binding-site of inactive [4FeH] protein exposes a relatively large water-accessible surface so that oxygen species likely can reach and damage the [4Fe4S] cluster^31^. This might explain the loss of the maturation competence of the [4FeH] proteins. It remains to be shown whether O_2_-exposure produces a [3Fe4S] species, which can be reactivated, or other detrimental degradation reactions at the cofactor or the protein scaffold. However, the partial surface accessibility of the [4Fe4S] cluster also in HYDA1[6FeH] may suggest that reaction steps are involved in inactivation that are independent of the [2FeH] site.

Our structural characterization revealed that the metric parameters of lyophilized HYDA1[6FeH] derived by *in vitro* maturation of HYDA1[4FeH] from *E. coli* with 2Fe_adt_ closely resemble the parameters of HYDA1[6FeH] from *C. acetobutylicum in vivo* maturated via HYDE,F,G^10^,^20^,^21^,^27^,^28^. This indicated an intact structure of the H-cluster in the *in vitro* maturated holoenzyme, which was fully preserved after lyophilization. The XAS data further show that an intact H-cluster was still present after 15 min exposure of lyophilized HYDA1[6FeH] to pure O_2_ gas. However, the observed slight geometry changes in the cofactor are compatible with O_2_-binding and single-electron oxidation of the complex, meaning that O_2_ may have partially reacted with the H-cluster in the lyophilized [FeFe]-hydrogenase. Binding of a single O_2_ molecule at Fe_d_ has been proposed to initiate the oxidative H-cluster degradation reactions^20^,^21^. Furthermore, this process may crucially depend on specific protonation events of formed reactive oxygen species^55^.

The removal of bulk and protein-bound water from the [FeFe]-hydrogenase by lyophilization may be expected to cause a rupture of the proton transfer pathway leading from the protein surface to the active site. For lyophilized [NiFe]-hydrogenases it has been shown that H/D-isotope exchange activity, which depends on a functional proton transfer pathway, can only occur at dramatically lowered rates^56^,^57^. Accordingly, the absence of protonation of reactive oxygen species formed at the H-cluster after the binding of O_2_ provides a plausible explanation for the O_2_-resistance of the lyophilized [FeFe]-hydrogenases. Thereby, the H-cluster may be trapped after the first reaction step when O_2_ is still bound to Fe_d_. This situation would prevent binding and reaction of further O_2_, which in solution leads to partial or complete H-cluster degradation^21^,^40^. This mechanism implies that the degradation process may continue as soon as proton transfer is re-established, thereby explaining the inactivation of the lyophilized [FeFe]-hydrogenases at increasing rehydration levels. That lyophilized and O_2_-exposed HYDA1 was fully active in anaerobic solution suggests that the oxygen species bound to the H-cluster was safely removed by dissolution under reducing conditions of the protein, thus preventing irreversible O_2_-inhibition.

In conclusion, our biochemical and spectroscopic experiments on lyophilized [FeFe]-hydrogenases provide clear evidence that water plays an important role in the O_2_-induced inactivation and degradation reactions at the H-cluster. These reactions are effectively prevented in the lyophilized proteins, preserving full activity even after long-term exposure to pure O_2_. Lyophilized and thereby highly up-concentrated and water-free [FeFe]-hydrogenase protein samples are particularly suitable for advanced spectroscopic analyses resulting in superior signal quality. The catalytic competence and structure of the active-site H-cluster in the enzymes is unaffected by lyophilization and rehydration. Lyophilization of [FeFe]-hydrogenases thus is a straightforward method to conserve the versatile chemical properties of these fascinating H_2_-catalysts.

## Methods

### Organisms and growth conditions

*Escherichia coli* strain DH5α MCR was used for the cloning procedures of the expression constructs described below. Heterologous expression of the [4FeH] (apo-) and holo- protein [6FeH] of *Chlamydomonas reinhardtii* HYDA1 as well as of *Clostridium pasteurianum HYDA* (CPI) was carried out in *E. coli* strain BL21 (DE3) Δ*iscR*^58^ according to the previously published procedure^13^,^14^,^32^.

### Cloning of HIS_6_-tagged *C. reinhardtii* HYDA1 and *C. pasteurianum* CPI variants

*E. coli* codon optimized cDNA of *HYDA1* or gDNA of *CPI* both fused at their 3′end to a short DNA sequence that encodes a Strep-Tag II affinity tag and cloned into pET21b^14^,^32^ were used as templates for the respective amplifications with Pfu DNA polymerase. For the PCR based C-terminal fusion of the HIS_6_-tag sequence to HYDA1 the oligonucleotides 5′-CTCGATCCCGCGAAATT-3′ (forward), binding upstream of the *HYD* genes and 5′-CGG**GTCGAC**TTA*GTGATGATGGTGGTGATG*GGCTGATTTTTTTTCATCTTTTTC-3′ (reverse) were used. For the C-terminal fusion of HIS_6_ to CPI the same forward primer, but the CPI specific reverse primer 5′-CGG**GTCGAC**TTA*GTGATGATGGTGGTGATG*GGCTGATTTTTTATATTTAAAGTGCAGGATTTCATGGGCACGACCTTC-3′ was used. Bold letters indicate the restriction site for *Sal*I which together with *Nhe*I was used for cloning, while bold italic letters indicate the HIS_6_-tag coding sequence. The amplified constructs were again cloned into the expression vector pET21b using *Nhe*I and *Sal*I restriction sites and the resulting plasmids verified by DNA-sequencing. The resulting constructs pET21b-HydA1opt-6H and pET21b-CpIopt-6H facilitated a regulated expression via the IPTG inducible lac-promoter.

### Heterologous production and purification of recombinant HYDA1 and CPI

*E. coli* BL21 (DE3) ΔiscR expression strains^58^ were generated via electrotransformation with constructs pET21b-HydA1opt or pET21b-CpIopt encoding either HIS_6_- or Strep-tag II fusion proteins, commonly without, but also with maturation factors HYDE,F,G,X encoded on a second expression vector pACYCDuet-1, for determining the specific activity of HYDA1[4FeH]+[2FeH] (Supplementary Tab. S1)^32^. Cultivation and enzyme purification via Strep-tag II affinity tag was performed as previously described^14^,^32^. For purification with Ni-NTA IMAC, *E. coli* cells, resuspended in 100 mM Tris-HCl (pH 8), plus 2 mM sodium dithionite (NaDT) were lysed by ultrasonication (five times 30 s; output 25; Branson Sonifier 250). Cell debris was removed via centrifugation (60 min, 200.000 × *g*, 4 °C). For IMAC purification the supernatant was diluted with an aqueous solution containing imidazole and NaDT to final concentrations of 50 mM Tris-HCl (pH 8), 10 mM imidazole, and 2 mM NaDT. The resulting solution was passed through a sterile filter (pore size 0.2 μm). Afterwards the filtrate was loaded on a 4 mL gravity flow Ni-NTA fast-flow column pre-equilibrated with 50 mM Tris-HCl (pH 8), supplemented with 10 mM imidazole and 2 mM NaDT. After washing with 50 mM Tris-HCl (pH 8) containing 20 mM imidazole and 2 mM NaDT the protein was eluted from the column using 100 mM imidazole. Purified proteins were stored at −80 °C until further use after concentrating with Amicon centrifugal filter units (30 kDa cutoff). Protein concentration was determined via spectrophotometry (NanoDrop, Peqlab) at λ = 280 nm. Molecular weight and purity of the eluated protein fractions were analyzed via denaturing sodium dodecylsulfate polyacrylamide gel electrophoresis (SDS-PAGE) using Coomassie brilliant blue staining according to standard techniques (Supplementary Fig. S1b). HYDA1[6FeH] and CPI[6FeH] samples were prepared by *in vitro* maturation of concentrated HYDA1[4FeH] and CPI[4FeH] expression products by addition of the chemically synthesized 2Fe_adt_ cofactor with subsequent purification using a desalting column as described previously^14^.

### Lyophilization of [FeFe]-hydrogenases

The hydrogenases, either [4FeH] or [6FeH] proteins supplemented with 100 mM NaDT, were dispensed in PCR-tubes with a pierced lid. Sample tubes were anaerobically frozen in a round-bottom flask filled with liquid nitrogen and subsequently attached to a freeze-dryer (LyoVac GT 2; Leybold - Heraeus) for 24 h. Protein concentration ranged from 1 to 110 mg mL^−1^ for HYDA1 and was 0.5 mg mL^−1^ for CPI. After water sublimation the sample-containing flask was sealed airtight under low pressure and the lyophilized samples were reintroduced into the anaerobic tent. To avoid sample humidification, the proteins powder was hermetically sealed and stored at −80 °C until further use. The temporary cryogenic sample storage did not affect the protein properties in any way.

### O_2_-exposure of lyophilized [FeFe]-hydrogenases

The lyophilized proteins were continuously gassed with dry pure O_2_ gas at room temperature for 1–8 h. For 24 h O_2_-exposure, samples obtained after O_2_-gassing for 8 h were incubated for another 16 h in an atmosphere of dry pure O_2_ in airtightly sealed suba vials. After the O_2_-treatments, the samples were purged with argon for 10 min before activity measurements were carried out.

### Exposure of lyophilized [FeFe]-hydrogenases to defined moisture levels and O_2_

Humidified O_2_ gas was prepared by filling the headspace of an airtight 20 mL suba vial containing 3 mL of 100 mM potassium phosphate buffer (pH 6.8) with pure O_2_ gas followed by a subsequent incubation over night at 80°C in a heating cabinet. For exposure of lyophilized hydrogenases to oxygen gas with increasing levels of humidity, protein aliquots were first flushed for 15 min with dry pure O_2_ gas. Afterwards, defined volumes of the water-saturated O_2_ gas were injected into airtight sealed sample-containing vials. Protein samples were exposed to the humidified O_2_-atmosphere for 30 min at room temperature. Immediately before determining the specific H_2_-evolution activity the sample vials were anaerobized by flushing with argon for 15 min.

### O_2_-exposure of [FeFe]-hydrogenases in solution

The two purified [FeFe]-hydrogenases from *C. reinhardtii* and *C. pasteurianum* either with or without the [2FeH] subsite were exposed for 15 min to different concentrations of O_2_. Therefore, solutions of 100 mM potassium phosphate pH (6.8) supplemented with 2 mM NaDT and 6.33 nM of the particular enzyme were flushed first with argon for 3 min, then different volumes of dry pure O_2_ gas were injected into the airtight sealed reaction tubes, and the samples were incubated for 15 min at 37 °C in a shaking water bath. For anaerobization, the samples were purged with argon for 5 min and specific H_2_-evolution activities were determined immediately.

### *In vitro* H_2_-evolution activity assay

For determining H_2_-evolution activities of [FeFe]-hydrogenase samples, the previously described *in vitro* activity assay was used^59^. To evaluate the maturation competence and thus integrity of hydrogenase apoenzymes ([4FeH]), the proteins were mixed with a 10-fold excess of the synthetic 2Fe_adt_ complex immediately prior to activity determination^14^.

### Preparation of samples for XAS on lyophilized HYDA1

HYDA1[4FeH] expressed in *E. coli* was purified and either used as prepared or after *in vitro* maturation as HYDA1[6FeH]. Samples of HYDA1[4FeH] and HYDA1[6FeH] were concentrated to 2.2 mM protein and NaDT was added to a final concentration of 100 mM. 16 μL sample volume was injected under anoxic conditions into Kapton-covered acrylic-glass sample holders using a Hamilton syringe^21^. Sample holders loaded with 2 mM of [FeFe]-hydrogenase protein were immediately frozen in a round-bottom flask containing liquid nitrogen and attached to the freeze-dryer for 24 h as described above. Afterwards the lyophilized samples were immediately frozen or exposed for 15 min to dry pure O_2_ gas and flushed thereafter with argon for 5 min before storage at −80 °C. Storage of the samples at −80 °C was done in a plastic zip bag, enclosed in a 50 mL falcon tube filled with Silica gel 60 to exclude any humidity. In addition, lyophilized HYDA1[6FeH] samples in XAS sample holders were exposed to air at ambient humidity for ~5 days prior to the XAS experiments.

### X-ray absorption spectroscopy

XAS at the Fe K-edge was carried out at beamline KMC-1 at BESSY (Helmholtz Zentrum für Materialien und Energie Berlin) with the storage ring operated in top-up mode (200 mA). A Si[111] double-crystal monochromator was used for incident energy scanning and a 13-element energy-resolving germanium detector (Canberra) was used for Fe X-ray fluorescence detection (shielded by 10** **μM Mn foil against scattered X-rays) in a standard XAS set-up^25^. The spot size on the sample was set by slits to about 5 (horizontal) × 0.5 (vertical) mm^2^. Samples were held in a liquid-helium cryostat (Oxford) at 20 K. The absorption spectrum of an Fe foil was measured in parallel and its first inflection point at 7112 eV was used for energy calibration^26^,^28^. Up to 4 XAS scans of ~30 min duration were carried out per sample (1 scan per spot) and dead-time corrected spectra were averaged, normalized, and EXAFS oscillations were extracted (*E*_0_ = 7112 eV) as previously described^25^. Fourier-transform (FT) calculation (using cos windows extending over 10% at both *k*-range ends) and least-squares fit analysis of unfiltered EXAFS spectra^25^ were carried out using in-house software^25^. Phase functions were calculated by FEFF7^60^ (*S*_0_^2^ = 0.9; *E*_0_ was refined to 7115 ± 2 eV in the EXAFS fits).

## Additional Information

**How to cite this article**: Noth, J. *et al.* Lyophilization protects [FeFe]-hydrogenases against O_2_-induced H-cluster degradation. *Sci. Rep.* **5**, 13978; doi: 10.1038/srep13978 (2015).

## Supplementary Material

Supplementary Information

## Figures and Tables

**Table 1 t1:** EXAFS simulation parameters of lyophilized HYDA1 proteins^a^.

		**N [per Fe ion]**/**R [Å]**/**2σ**^**2**^ **×** **10**^**3**^ **[Å**^**2**^]					**R_F_ [%]**
HYDA1:		Fe-C/O	Fe-S	Fe-Fe, [2FeH]	Fe-Fe, [4FeH]	Fe^…^O/N	
[4FeH]	−O_2_	–	4/2.28/7	–	3/2.73/10	–	9.0
	+O_2_	–	4/2.28/7	–	3/2.73/10	–	12.2
[6FeH]	−O_2_	1/1.91/5^#^	3.5/2.27/10	0.5/2.55/2^#^	2/2.72/7	1/3.00/5^#^	12.5
	+O_2_	1/1.87/5^#^ 0.82^*^/1.86/5^#^	3.5/2.26/9 3.65^*^/2.26/10^&^	0.5/2.58/2^#^ 0.5/2.57/2^#^	2/2.71/10 1.87^*^/2.71/7^&^	1/3.05/5^#^ 1/3.07/5^#^	14.4 14.7
	+O_2_ +H_2_O	3.07^*^/2.04/5^#^	1.73^*^/2.29/10^&^	0.05^*^/2.55/2^#^	0.39^*^/2.71/7^&^	0.34^*^/3.18/5^#^	13.8

^a^N, coordination number; R, interatomic distance; 2σ^2^, Debye-Waller parameter; R_F_, fit error sum calculated for reduced distances of 1–3 Å^25^. Data correspond to EXAFS spectra in Fig. 4b. Fit restraints: N-values were fixed to the given numbers in the fits representing the expected coordination numbers for the [4Fe4S] cluster in HYDA1[4FeH] or the approximate N-values for a complete H-cluster in HYDA1[6FeH]^21^,^28^, ^*^except for N-values that were allowed to vary in the fits; ^&^the same 2σ^2^-values were used for respective fits of spectra plus or minus O_2_; ^#^2σ^2^ values that were fixed in the simulations. For [6FeH] +O_2_, the fit result for unrestricted N-values (*) emphasizes that the Fe-CO/N and Fe-S bonds and the Fe-Fe interactions in the H-cluster are fully preserved in the presence of O_2_.
