# Supplementary material for: Lyophilization protects [FeFe]-hydrogenases against O2-induced H-cluster degradation
Source: Sci Rep. 2015 Sep 14;5:13978. doi: 10.1038/srep13978 (PMC4568494; doi:10.1038/srep13978)
Supplement: Supplementary Information [file srep13978-s1.pdf]

Supplementary Information

Lyophilization protects [FeFe]-hydrogenases against O_2-induced H-cluster degradation

Jens Noth[1], Ramona Kositzki[2], Kathrin Klein[3], Martin Winkler[1], Michael Haumann[2] and Thomas Happe[1*]

[1]Ruhr-Universität Bochum, Fakultät für Biologie und Biotechnologie, Lehrstuhl für Biochemie der Pflanzen, AG Photobiotechnologie, 44801 Bochum, Germany

[2]Freie Universität Berlin, Institut für Experimentalphysik, 14195 Berlin, Germany

[3]Ruhr-Universität Bochum, Fakultät für Chemie und Biochemie, Anorganische Chemie I – Bioanorganische Chemie, 44801 Bochum, Germany

[*]*Correspondence to:*

Prof. Thomas Happe, Ruhr-Universität Bochum, Fakultät für Biologie und Biotechnologie, Lehrstuhl für Biochemie der Pflanzen, AG Photobiotechnologie, Universitätsstrasse 150, 44801 Bochum, Germany

Phone: +49 234 32 27026, Fax: +49 234 32 14322, Email: thomas.happe@rub.de

This file includes:

Supplementary Table S1: ***Comparison of HYDA1 protein purification yields***

Supplementary Figure S1: ***Purification and activity measurements of HIS_6- and STII-tagged HYDA1 and CPI***

Supplementary References

Supplementary Table S1. Comparison of HYDA1 protein purification yields.[a]

Expression host	Cluster occupation	Purification tag	Yield [mg L^{-1}]	Spec. activity [µmol $H_2 \cdot mg^{-1} \cdot min^{-1}$]	Reference
C. reinhardtii	[4FeH] + [2FeH]	w/o	$3.2 \cdot 10^{-4}$	360	Roessler and Lien, 1984
C. reinhardtii	[4FeH] + [2FeH]	w/o	0.07	935	Happe and Naber, 1993
E. coli	[4FeH] + [2FeH]	STII	0.9	150	King et al, 2006
S. oneidensis	[4FeH] + [2FeH]	STII	0.45	700	Sybirna et al, 2008
C. acetobutylicum	[4FeH] + [2FeH]	STII	1.0	625	v. Abendroth et al, 2008
E. coli	[4FeH] + [2FeH]	STII	30	641	Kuchenreuther et al, 2010
E. coli	[4FeH] + [2FeH]	HIS_6	45	660	This work
E. coli	[4FeH]<br>[4FeH] + [$2FeH_{adt}$]	HIS_6	90	0<br>950	This work

[a]Specific activities in the present work were determined using the sodium-dithionite-driven hydrogen-production-assay with methyl viologen as an artificial electron mediator. Activities in ref.[1] were determined using 10 µM of ferredoxin for electron shuttling instead and the total reported yield had a purity of approximately 40%. HYDA1[4FeH] was *in vitro* maturated with $2Fe_{adt}$ prior to activity determination[2].

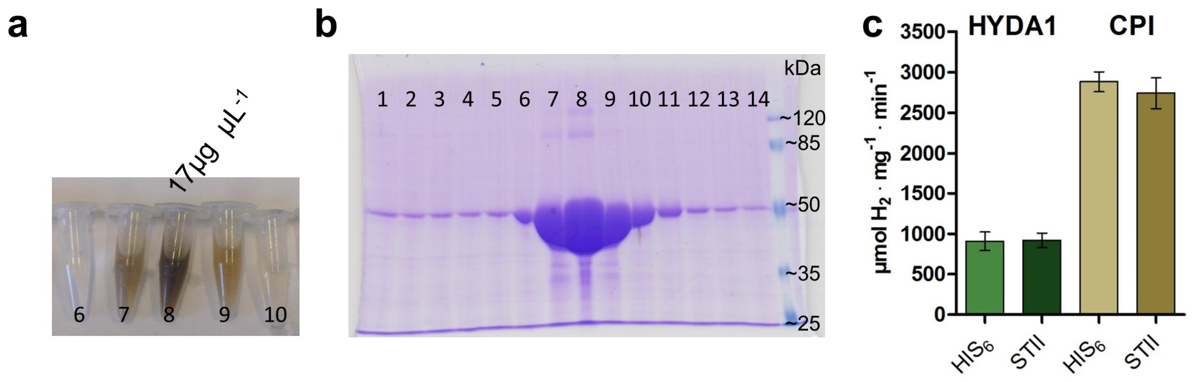

Supplementary Figure S1. Purification and activity measurements of HIS_6- and STII-tagged HYDA1 and CPI. A Ni-NTA gravity-flow column with a volume of 4 mL was used to purify 45 mg of HYDA1[4FeH]-HIS_6 from 0.5 L of *E. coli* culture. (A) Photograph of the main [FeFe]-hydrogenase elution fractions (6-10), the dark-brown fraction 8 with a volume of 1.1 mL has a concentration of 17 µg protein μL^{-1} (340 µM). (B) The Coomassie-stained SDS-PAGE (10%) shows one significant protein band at a molecular weight of 50 kDa. Each lane (1-14) was loaded with 5 µL of protein eluate. (C) Comparison of the specific activities of Strep-tag II (STII) and HIS_6-tag purified hydrogenase proteins HYDA1[4FeH] and CPI[4FeH] were *in vitro* activated with $2Fe_{adt}$[2] prior to the activity assay. 1 µg HYDA1 or 0.5 µg CPI was used in the activity assays, each data point represents the mean of 3 measurements, error bars give standard deviations.

Supplementary references

1. Roessler, P. G. & Lien, S. Purification of hydrogenase from *Chlamydomonas reinhardtii*. *Plant Physiol.* **75**, 705-709 (1984).
2. Esselborn, J., *et al.* Spontaneous activation of [FeFe]-hydrogenases by an inorganic [2Fe] active site mimic. *Nat. Chem. Biol.* **9**, 607-609 (2013).
3. Happe, T. & Naber, J. D. Isolation, characterization and N-terminal amino acid sequence of hydrogenase from the green alga *Chlamydomonas reinhardtii*. *Eur. J. Biochem.* **214**, 475-481 (1993).
4. King, P. W., Posewitz, M. C., Ghirardi, M. L. & Seibert, M. Functional studies of [FeFe] hydrogenase maturation in an *Escherichia coli* biosynthetic system. *J. Bacteriol.* **188**, 2163-2172 (2006).
5. Sybirna, K., *et al. Shewanella oneidensis*: a new and efficient system for expression and maturation of heterologous [Fe-Fe] hydrogenase from *Chlamydomonas reinhardtii*. *BMC Biotechnol.* **8**, 73 (2008).
6. von Abendroth, G., *et al.* Optimized over-expression of [FeFe] hydrogenases with high specific activity in *Clostridium acetobutylicum*. *Int. J. Hydrogen Energ.* **33**, 6076-6081 (2008).
7. Kuchenreuther, J. M., *et al.* High-yield expression of heterologous [FeFe] hydrogenases in *Escherichia coli*. *PloS One* **5**, e15491 (2010).
